# Weak hy­dro­gen bonding in the structures of three double-acyl­ated amino­anti­pyrines

**DOI:** 10.1107/S2053229625009581

**Published:** 2025-11-10

**Authors:** Lina Mardiana, Afnan B. Al Abdali, Michael J. Hall, Hamad H. Al Mamari, Paul G. Waddell

**Affiliations:** a Chemistry – School of Natural and Environmental Sciences, Bedson Building, Newcastle University, Newcastle upon Tyne, NE1 7RU, United Kingdom; b Department of Chemistry, Universitas Indonesia, Depok, Jawa Barat, 16424, Indonesia; c Indicatrix Crystallography, Bedson Building, Newcastle University, Newcastle upon Tyne, NE1 7RU, United Kingdom; d Department of Chemistry, College of Science, Sultan Qaboos University, PO Box 36, Al Khoudh 123, Muscat, Sultanate of Oman; J-PARC Center, Japan Atomic Energy Agency, Japan

**Keywords:** crystal structure, aryl­ation, crystal packing, weak hy­dro­gen bonds, π-inter­actions, ENaCt, 4-amino­anti­pyrine

## Abstract

The structures of three doubly-acyl­ated 4-amino­anti­pyrine (AP) com­pounds where the aryl substituent is varied are reported and analysed in terms of their relative conformation, inter­molecular inter­actions and overall packing; these are *N*-(1,5-dimethyl-3-oxo-2-phenyl-2,3-di­hydro-1*H*-pyrazol-4-yl)-4-methyl-*N*-[(4-methyl­phen­yl)carbon­yl]benzamide, C_27_H_25_N_3_O_3_, *N*-(1,5-dimethyl-3-oxo-2-phenyl-2,3-di­hydro-1*H*-pyrazol-4-yl)-*N*-[(furan-2-yl)carbon­yl]furan-2-carbox­am­ide, C_21_H_17_N_3_O_5_, and *N*-(1,5-dimethyl-3-oxo-2-phenyl-2,3-di­hydro-1*H*-py­ra­zol-4-yl)-*N*-[(thio­phen-2-yl)carbon­yl]thio­phene-2-carboxamide, C_21_H_17_N_3_O_3_S_2_. The com­pounds were crystallized using the encapsulated nanodroplet crystallization (ENaCt) protocol. Where previous singly-acyl­ated AP com­pounds pro­duced structures with obvious classical hy­dro­gen-bonding motifs, the doubly-acyl­ated derivatives lack classical donors and therefore exhibit weak C—H hy­dro­gen bonds and inter­actions involving the π-system. All three AP com­pounds form bifurcated C—H⋯O inter­actions having either dimer or chain motifs, with the other structure-directing inter­actions being dependant on the nature of the aryl substituent.

## Abstract 2

The structures of three doubly-acyl­ated 4-amino­anti­pyrine com­pounds where the aryl substituent is varied are reported and analysed in terms of their relative conformation, inter­molecular inter­actions and overall packing. The com­pounds were crystallized using the encapsulated nanodroplet crystallization (ENaCt) protocol.

## Introduction

The 4-amino­anti­pyrine (AP) moiety has been studied extensively due to its historic use as analgesic/anti-inflammatory medi­cation (Ampyrone), albeit with potentially serious side effects, with safer variants (*e.g.* Metamizole) typically involving modification of the pendant amino group (Brogden, 1986 ▸). Although APs are still of inter­est due to their biological activity (Kurdekar *et al.*, 2012 ▸), more recently, pyrazolo­nes, such as AP, have been employed as *N*,*O*-bidentate directing groups, finding use in Ru-catalysed C(*sp*^2^)—H bond aryl­ation reactions (Al Mamari *et al.*, 2021 ▸, 2024 ▸).

In addition, there have been a great many structural studies of com­pounds bearing the AP moiety (Singh *et al.*, 2020 ▸; Erturk, 2020 ▸; Shankar *et al.*, 2023 ▸), with analysis of the supra­molecular structure providing insights into their potential use in non-linear optics (Montalvo-González & Ariza-Castolo, 2003 ▸; Arumugam *et al.*, 2023 ▸). These studies have often focused on AP derivatives with obvious strong hy­dro­gen bonding, where common motifs were identified, and some C—H⋯O, C—H⋯π and π–π inter­actions are also observed (Mnguni & Lemmerer, 2015 ▸; Narayana *et al.*, 2016 ▸).

During the preparation of acyl­ated AP derivatives for use in directed Ru-catalyzed C—H aryl­ation chemistry (Al Mamari *et al.*, 2021 ▸), over-acyl­ation of the pendant amino group was observed in a number of cases. This led to the formation of a set of double-acyl­ated APs (com­pounds **1**–**3**; Scheme 1), con­taining *para*-toluoyl, 2-furoyl and 2-thenoyl groups, respectively, which are the focus of this study.

One consequence of this double acyl­ation is that, com­pared to where only a single acyl­ation occurs, the com­pounds lack classical hy­dro­gen-bond donors. As a result, without the formation of these rather obvious inter­actions, the packing is likely to be dominated by weak hy­dro­gen bonds incorporating C—H donor protons and/or inter­actions involving the π-systems of the aromatic substituents.

To com­plement previous studies into hy­dro­gen-bonding net­works in these com­pounds, this work looks into which inter­actions form in the absence of classical hy­dro­gen bonds and how this affects the packing in a series of related doubly-acyl­ated AP mol­ecules where the aryl substituent is varied.

## Experimental

### Synthesis

Double-acyl­ated AP derivatives were prepared through the reaction of 2.5–3.0 equivalents of the corresponding acyl chlo­ride with 4-amino­anti­pyrine, in the presence of an excess of Et_3_N, in CH_2_Cl_2_ at 0 °C for 17 h. Following work-up and purification, all three com­pounds were obtained in reasonable yield (Scheme 1 shows the structures of the double-acyl­ated AP derivatives, with the AP core structure highlighted in red). Detailed synthetic protocols can be found in the supporting information.
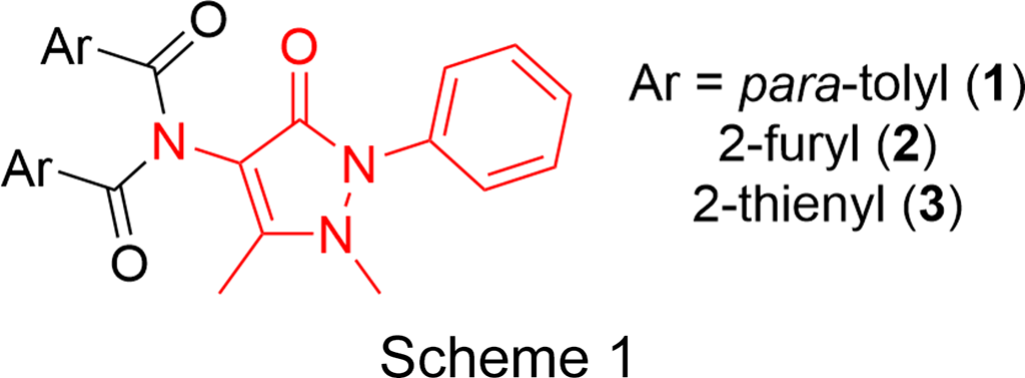


### Crystallization by ENaCt

Single crystals suitable for single-crystal X-ray diffraction (SCXRD) analysis were grown using a high-throughput solution-phase approach, known as encapsulated nanodroplet crystallization or ENaCt (Tyler *et al.*, 2020 ▸; Metherall *et al.*, 2023 ▸). ENaCt has been used successfully in the discovery of crystal forms of a wide range of small mol­ecules (Straker *et al.*, 2023 ▸; Metherall *et al.*, 2024 ▸), including recent applications in cocrystal and polymorph discovery (Metherall *et al.*, 2025 ▸; Weatherston *et al.*, 2025 ▸). Thus, near saturated solutions of com­pounds **1**–**3** were prepared, using 12 different solvents, through portionwise solvent addition to solid samples until dissolution was achieved. An SPT Labtech mosquito liquid handling robot was then used to dispense 50 nl droplets of these solutions into pre-dispensed 200 nl oil droplets, within a 96-well SWISSCI LCP glass plate. This resulted in 288 crystallization experiments per com­pound, with the combination of 12 solvents and four encapsulation oils (including no-oil conditions) resulting in 60 unique crystallization conditions. Plates were then sealed with a glass cover plate and crystallization monitored by cross-polarized optical microscopy. After 14 d, crystallization outcomes were recorded and suit­able single crystals retrieved for SCXRD analysis.

**Figure 1:**
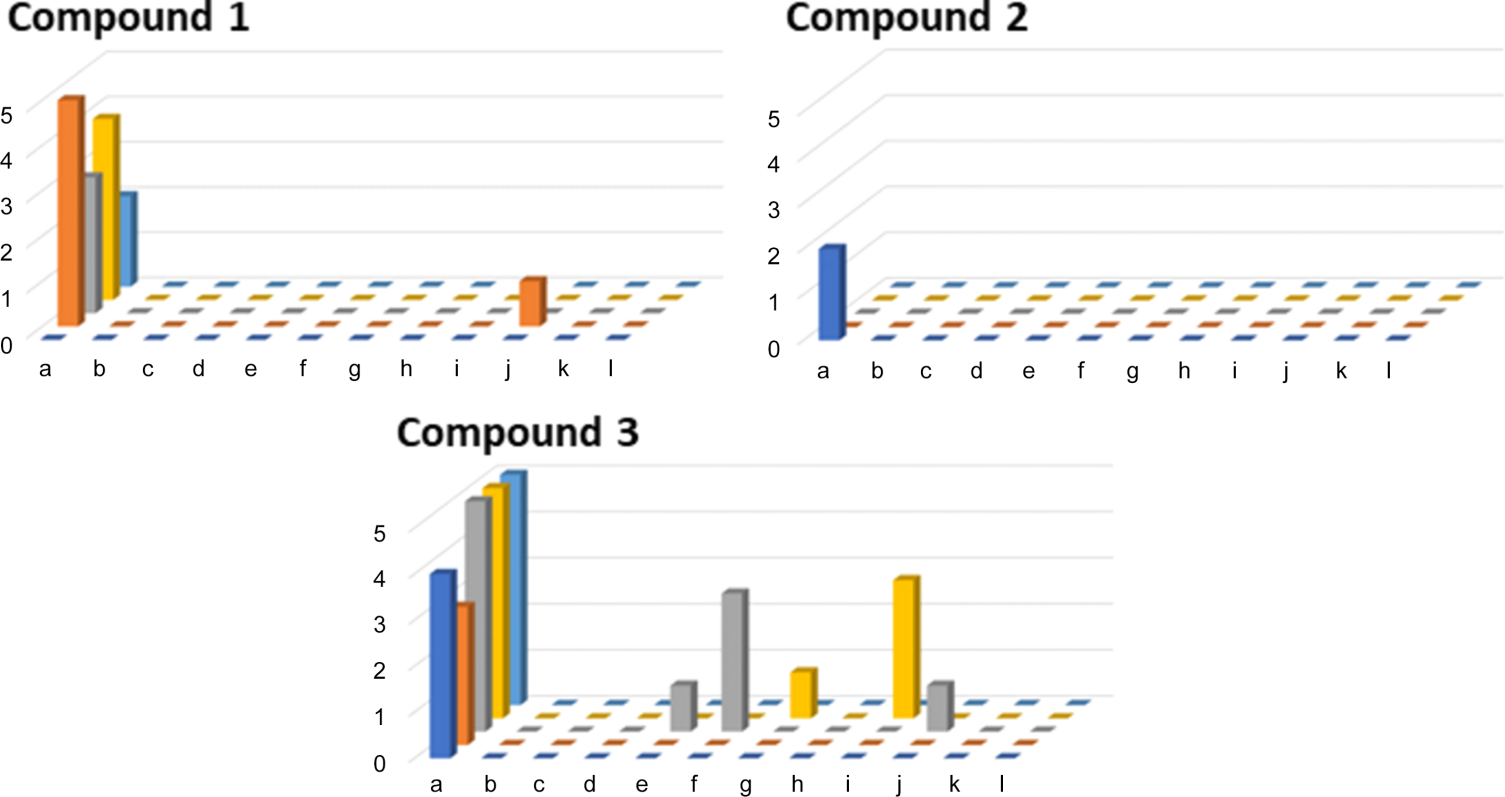
Experimental ENaCt outcomes for each com­pound, showing the total number of crystals identified as suitable for SCXRD *versus* experimental conditions {solvent (a = DMSO, b = DMF, c = MeOH, d = TFE, e = toluene, f = DCE, g = 2-MeTHF, h = 1,4-dioxane, i = EtOAc, j = MeCN, k = MIBK and l = NM) and oil [no oil (dark blue), PDMSO (orange), FC-40 (grey), FY (yellow) and MO (light blue)]}.

Compound **3** showed the highest levels of crystallinity in these experiments, with 31 from a total of 288 wells (11%) giving crystals likely suitable for SCXRD analysis. Next most crystalline was com­pound **1** (15 wells from 288, 5.2%), with com­pound **2** showing only 2 hits from the total screen (0.69%). Inter­estingly, all three com­pounds showed the best crystallization outcomes from ENaCt experiments using dimethyl sulfoxide (DMSO) as solvent, with only com­pound **3** showing significant ‘hits’ outside of this solvent (Fig. 1 ▸).

### SCXRD

**Figure 2:**
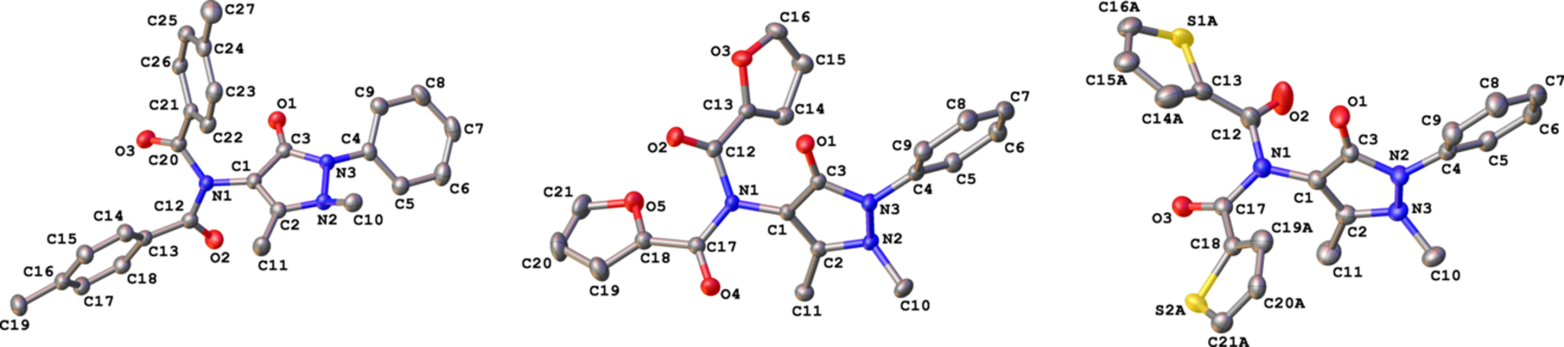
The crystal structures of **1**–**3** (left to right), with displacement ellipsoids drawn at the 50% probability level. H atoms have been omitted for clarity.

Crystal data, data collection and structure refinement details for **1**–**3** are summarized in Table 1 ▸. All structures (Fig. 2 ▸) were solved using *SHELXT* (Sheldrick, 2015*a* ▸) and refined by *SHELXL* (Sheldrick, 2015*b* ▸) using the *OLEX2* inter­face (Dolomanov *et al.*, 2009 ▸). All non-H atoms were refined anisotropically and H atoms were positioned with idealized geometry. The displacement parameters of the H atoms were constrained using a riding model with *U*_iso_(H) set to be an appropriate multiple of the *U*_eq_ value of the parent atom.

## Results and discussion

**Figure 3:**
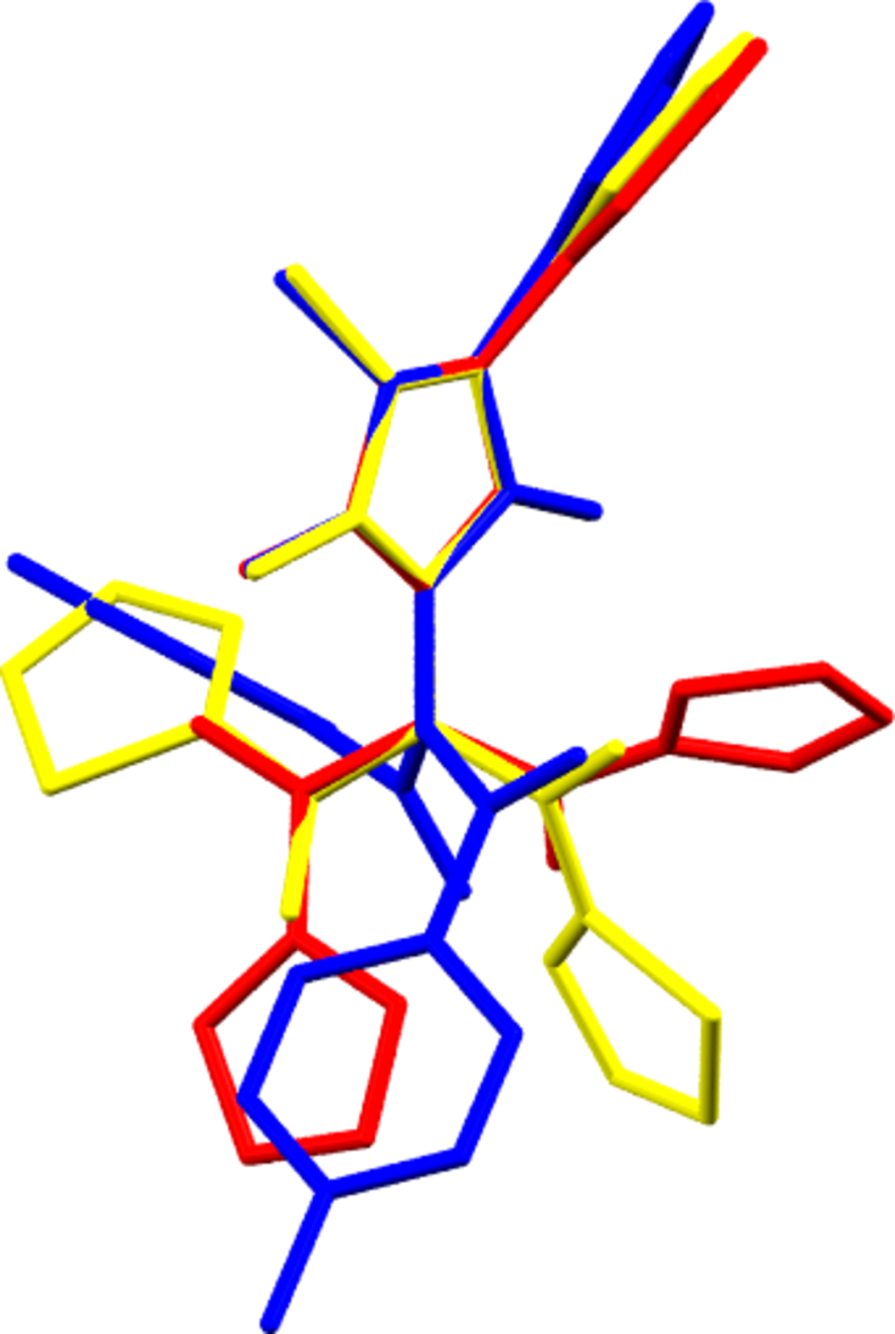
Overlay of the crystal structures of **1**–**3**.

The three structures of the double-acyl­ated AP com­pounds, though differing only in the identity of the aryl *R* group of the acid chloride starting material and crystallizing in the same space group (*P*2_1_/*c*), differed very starkly in terms of their conformation and packing. Each asymmetric unit comprises one molecule (*Z*′ = 1) and although the AP moiety is consistent across the three structures with respect to conformation, as only a slight variation in the angle of the phenyl group is observed, when the five-membered rings of all three molecules are overlayed, the difference in the conformation about the tertiary amine N atom between **1**, **2** and **3** becomes apparent (Fig. 3 ▸).

In all three structures, the conformation can be described with respect to three torsion angles corresponding to three distinct degrees of freedom. Firstly, by the orientation of the substituents relative to the asymmetrically-substituted five-membered ring, represented qu­anti­tatively by the C3—C1—N1—C12 torsion angle, and secondly, by the torsion angles about the amide bonds (Table 2 ▸).

Considering the C3—C1—N1—C12 torsion angle, the greater the steric bulk of the substituent (tolyl > thio­phenyl > furan­yl) the greater the value of the torsion angle: **1** > **3** > **2**. It is likely that this results from the minimization of steric inter­actions between the aforementioned substituent and the O1 atom and C11 methyl group of the AP moiety. In terms of the torsion angles about the amide bond, all three structures exhibit a similar pattern, with one acute and one obtuse angle. The values observed for **1** and **3** are essentially identical, whereas those of **2** are slightly shallower and are reversed relative to the other two structures, with the carbonyl group orientated in the opposite directions with respect to the AP moiety.

The variation in these torsion angles produces three very different conformations, which have a drastic effect on the packing in the structures, particularly in terms of the orientations of the aryl groups and the inter­actions between them.

**Figure 4:**
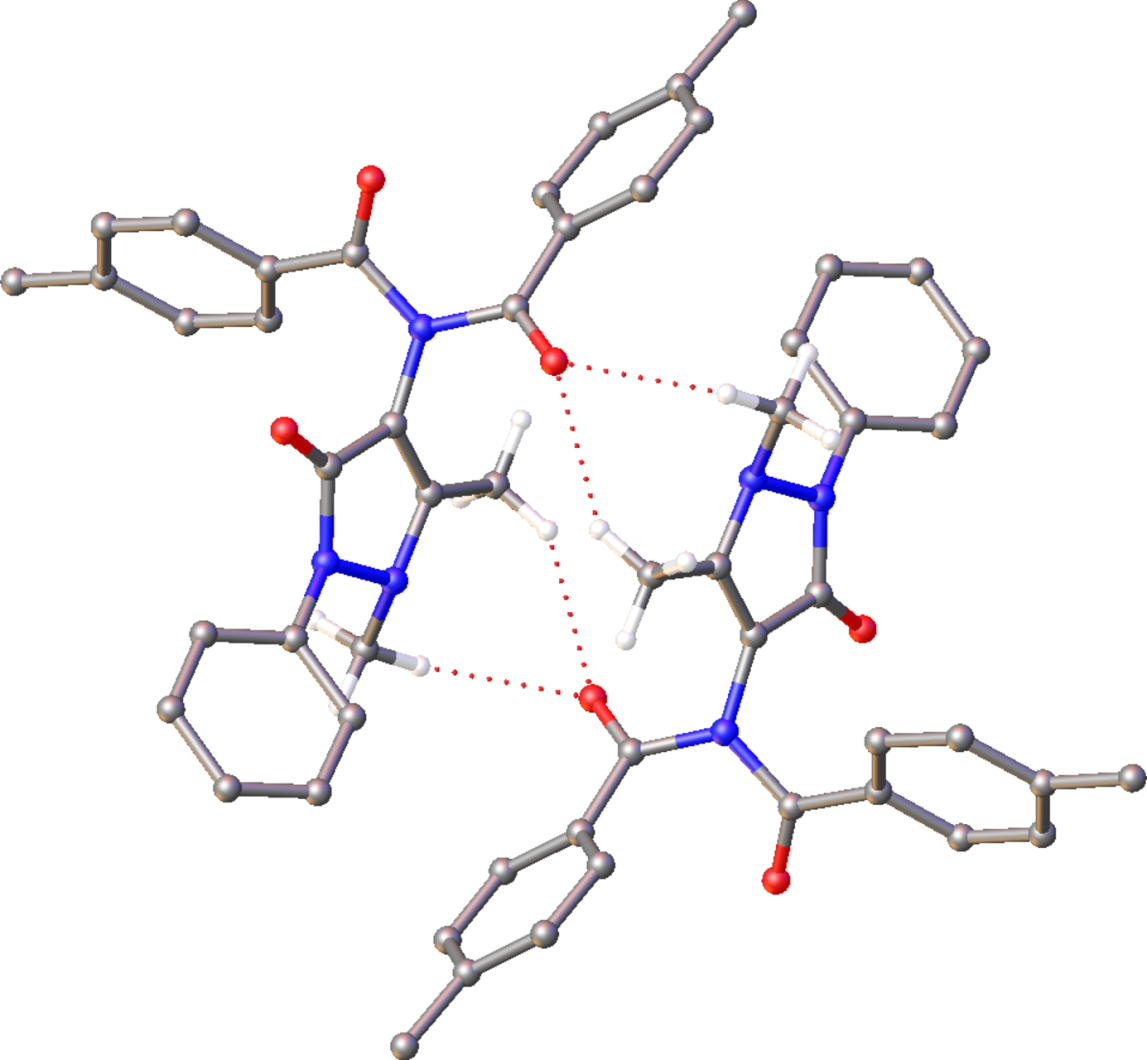
The weak hy­dro­gen-bonded dimer in the crystal structure of **1**. Hydrogen bonds are denoted by dashed lines and H atoms of groups not involved in hy­dro­gen bonding have been omitted for clarity.

The packing in the structure of **1** is unique among this group, as the mol­ecules crystallize as dimers formed of C—H⋯O hy­dro­gen bonds (Desiraju, 1991 ▸, 1996 ▸), where the two mol­ecules of the dimer are related by inversion symmetry (Fig. 4 ▸). The C—H⋯O inter­actions form as bifurcated hy­dro­gen bonds between the two methyl groups of the AP moiety and the carbonyl O atom of one of the aryl groups, with donor–acceptor distances of *ca* 3.3 Å (Table 3 ▸). As such, the structure is best described in terms of the packing of these dimer units.

This bifurcated C—H⋯O hy­dro­gen-bond motif is apparent in approximately 20% of AP structures in the Cambridge Structural Database (CSD; 70 out of 340; Groom *et al.*, 2016 ▸) and appears to be most prevalent among structures with no classical hy­dro­gen-bond donors (Montalvo-González & Ariza-Castolo, 2003 ▸; Singh *et al.*, 2020 ▸).

In addition to the weak hy­dro­gen bonding, the tolyl rings of adjacent dimer units are observed to arrange themselves in a face-to-face orientation, but, as the centroid–centroid dis­tances are not within the accepted range for π–π inter­actions (Avasthi *et al.*, 2014 ▸), the orientation of the rings is likely to be to minimize steric inter­actions.

**Figure 5:**
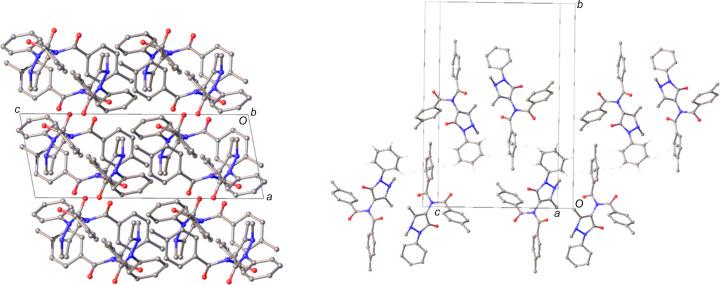
Views highlighting the layered structure (left) and the C—H⋯π hy­dro­gen-bonded chain (right) in the crystal structure of **1**. Hydrogen bonds are denoted by dashed lines, ring centroids as grey spheres and H atoms have been omitted for clarity with the exception of those of the phenyl groups involved in C—H⋯π bonding.

The crystal structure of **1** is observed to form layers of dimer units coplanar to the crystallographic (001) plane (Fig. 5 ▸). Between the layers, there appear to be edge-to-face inter­actions (Nishio, 2004 ▸; Brunner *et al.*, 2014 ▸) between the phenyl and tolyl groups (Table 3 ▸). These inter­actions link the dimer units to form a chain motif along the [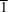
02] direction and, when considered along with the slightly longer intra-dimer contacts of the same type, form a continuous chain of C—H⋯π contacts in this direction.

**Figure 6:**
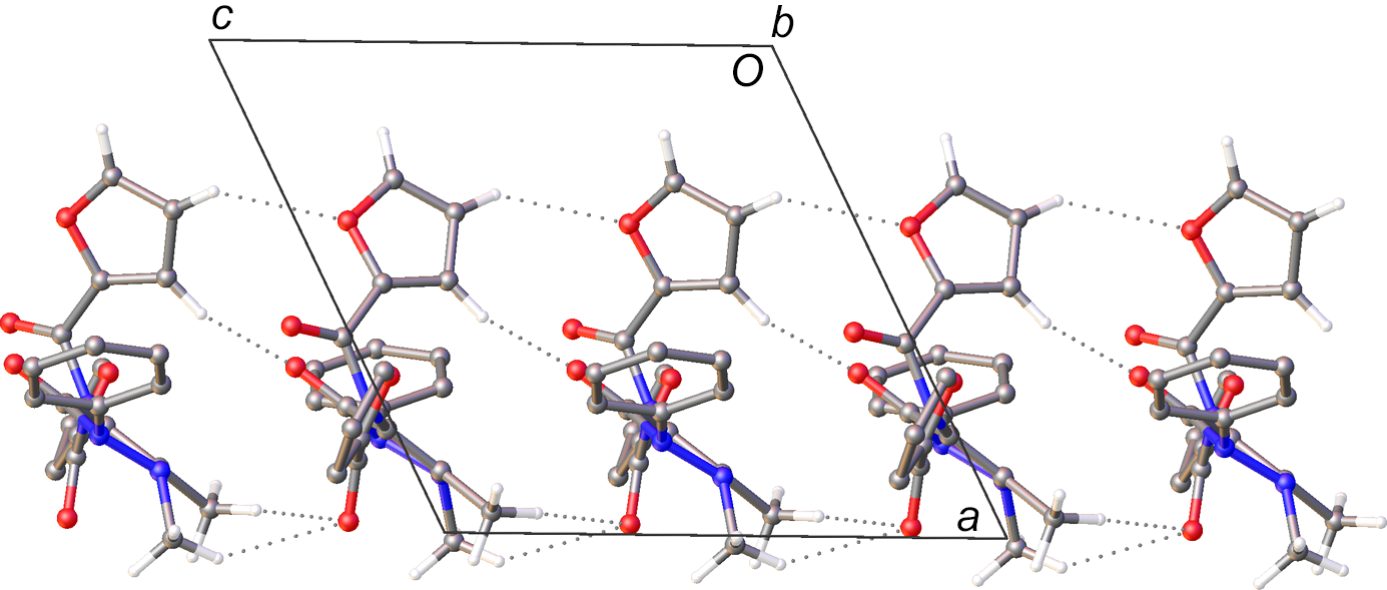
The C—H⋯O hy­dro­gen-bonded chain in the crystal structure of **2**. Hydrogen bonds are denoted by dashed lines and H atoms of groups not involved in hy­dro­gen bonding have been omitted for clarity.

The structure of **2** exhibits similar bifurcated C—H⋯O inter­actions to those observed for **1** (Table 4 ▸); however, in this case, instead of discrete dimers, these inter­actions form con­tinuous chains in the [001] direction, with each mol­ecule related to the next by the symmetry of the *c*-glide (Fig. 6 ▸). The formation of these chains is aided by inter­actions involving the furanyl group. This is observed in an additional C—H⋯O inter­action between a C—H hy­dro­gen-bond donor on the furanyl group and the carbonyl group of the AP moiety. The formation of this inter­action as a result of the introduction of the hy­dro­gen-bond-accepting furanyl group in contrast to the tolyl groups in **1** rationalizes both the formation of the chain and the mol­ecular conformation of **2** in the crystal structure.

There also appear to be C—H⋯O hy­dro­gen bonds of a similar distance between furanyl groups orientated along the [100] axis relative to the AP moiety that also extend along the length of the chain. Though the C—H⋯O hy­dro­gen-bond angle suggests that this inter­action is somewhat weaker than the Me⋯O inter­actions, it is still likely that it is having an effect on the orientations of the furanyl rings along the chain.

The phenyl and the other furanyl groups of the mol­ecule of **2** are orientated along the [010] direction relative to the AP moiety in an edge-to-face manner and, though one carbon–centroid distance is observed to be slightly below 4 Å, do not appear to form any salient inter­actions. It is likely that they are orientated to minimize steric inter­actions in much the same way as the tolyl and phenyl groups in the structure of **1**.

This notion of minimizing steric inter­actions is also clear in the orientation of the rings between the chains, as they also tend to exhibit an edge-to-face arrangement but with no inter­molecular distances that would indicate attractive inter­actions. In the [100] direction, the chains are connected by further C—H⋯O inter­actions between the furanyl group and the amide carbonyl group involved in the inter­actions that propagate along the chain.

**Figure 7:**
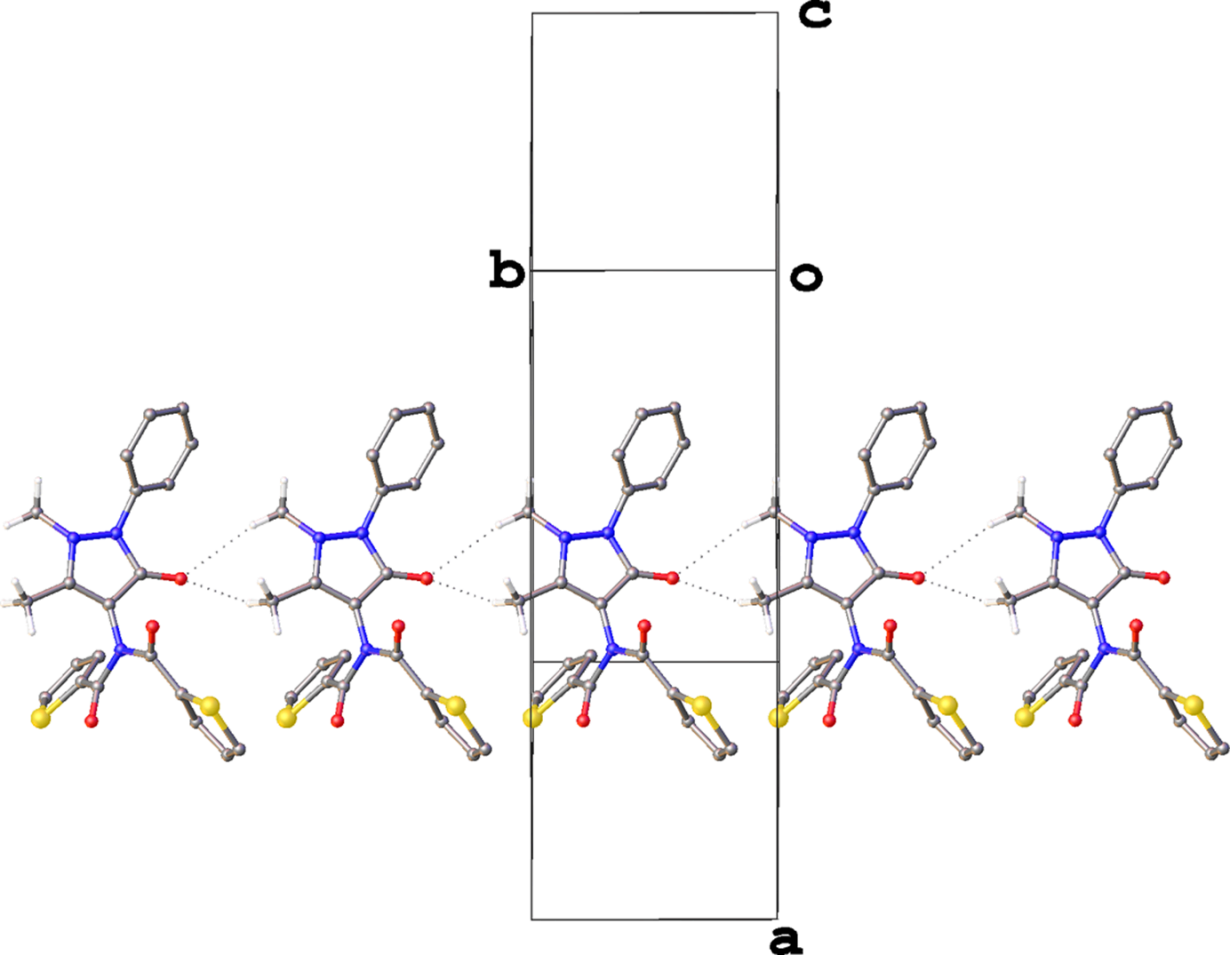
The C—H⋯O hy­dro­gen-bonded chain in the crystal structure of **3**. Hydrogen bonds are denoted by dashed lines and H atoms of groups not involved in hy­dro­gen bonding have been omitted for clarity.

The crystal structure of **3** combines features of both **1** and **2**. Like **1**, it has a layered structure, yet also exhibits chains of mol­ecules reminiscent of those in **2** (Fig. 7 ▸) formed of bifurcated C—H⋯O hy­dro­gen-bond inter­actions, though in this case the two distances are more uneven than those observed for either **1** or **2** (Table 5 ▸). Along the chain, each consecutive mol­ecule is related by pure translation symmetry in the [010] direction and, beyond the ubiquitous C—H⋯O inter­actions, there are no other potentially structure-directing inter­actions in this direction.

**Figure 8:**
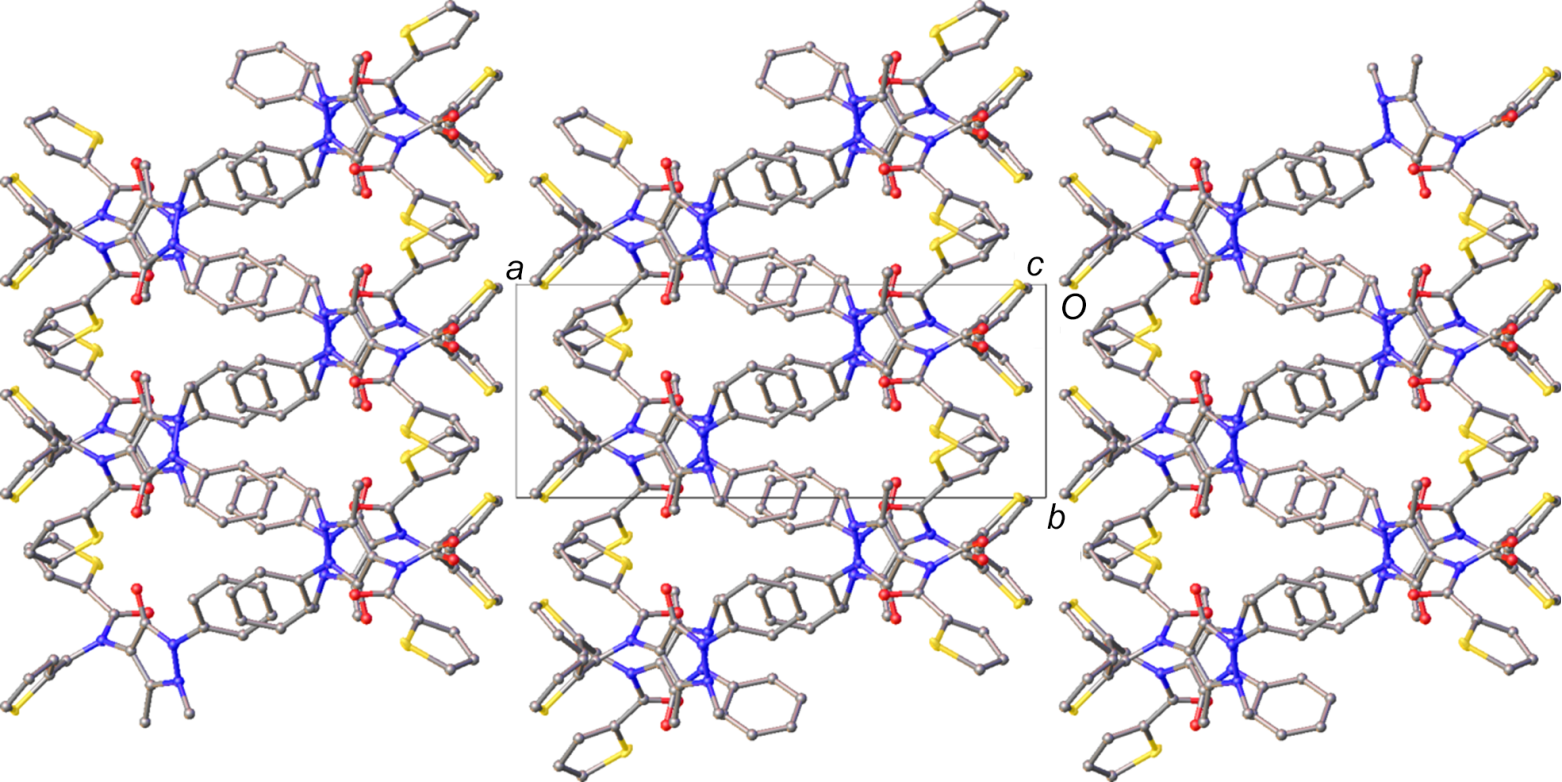
View highlighting the bilayers in the structure of **3**. H atoms have been omitted for clarity.

The layers observed in this structure are best described as bilayers coplanar with the crystallographic (100) plane (Fig. 8 ▸). The thio­phene rings of the mol­ecules of **3** are directed towards the boundary of the bilayers in an edge-to-face arrangement across the inter­face. The disorder in these rings indicates that there are likely no strong inter­actions across the layer boundary. Inter­estingly, the structure of **3** is the only one studied herein where the phenyl and aryl groups do not form motifs in which these rings alternate. In addition to the thio­phenes being directed toward the layer boundaries, the phenyl rings form an edge-to-face herringbone arrangement in the centre of the bilayer, such that the thio­phene and phenyl rings do not come into contact with each other.

## Conclusion

The three double-acyl­ated AP mol­ecules reported in this study all lack classical hy­dro­gen-bond donors and as such their crystal packing is directed by weak hy­dro­gen bonds incorporating C—H donors. As these inter­actions are weak, minor structural variations can have drastic effects on the crystal structures and this can be observed in the conformation and packing as a result of the change in aryl group (tolyl, furanyl or thio­phen­yl).

All three structures exhibit the same bifurcated C—H⋯O inter­actions between the methyl groups of the AP moiety and an amide carbonyl, but the symmetry relationship between the mol­ecules involved in this inter­action is different in each case. The larger tolyl group in **1** leads to the formation of a layered structure of discrete dimers where the packing is dictated by the steric bulk of the tolyl group and the need for unfavourable inter­actions between them to be minimized. In contrast, the smaller furanyl group of **2** has extra hy­dro­gen-bond-acceptor functionality, forming a chain motif with additional inter­actions between the furanyl groups. The thio­phenyl ana­logue, **3**, has an aryl group similar to the furanyl com­pound though slightly larger and with decreased hy­dro­gen-bond-acceptor ability. The result is a structure seemingly halfway between **1** and **2**, with inter­mediate torsion angles and both layers and chain motifs, though these differ from those of the other analogues forming as bilayers and a chain formed solely of bifurcated C—H⋯O inter­actions.

The insights provided here should surely be of inter­est to crystal engineers or anyone working in a field where solid-state structure has been shown to be important. As a case study, these serendipitous products show the effects of varying the substituents on mol­ecules of this kind can have on the packing in the absence of truly structure-directing inter­actions and classical hy­dro­gen bonds.

## Supplementary Material

Crystal structure: contains datablock(s) 3, 1, 2, global. DOI: 10.1107/S2053229625009581/oj3034sup1.cif

Structure factors: contains datablock(s) 3. DOI: 10.1107/S2053229625009581/oj30343sup4.hkl

Supporting information file. DOI: 10.1107/S2053229625009581/oj30343sup5.cml

Structure factors: contains datablock(s) 1. DOI: 10.1107/S2053229625009581/oj30341sup2.hkl

Supporting information file. DOI: 10.1107/S2053229625009581/oj30341sup6.cml

Structure factors: contains datablock(s) 2. DOI: 10.1107/S2053229625009581/oj30342sup3.hkl

Supporting information file. DOI: 10.1107/S2053229625009581/oj30342sup7.cml

Experimental details and NMR spectra. DOI: 10.1107/S2053229625009581/oj3034sup8.pdf

CCDC references: 2484901, 2484900, 2484899

## Figures and Tables

**Table 1:** Experimental details For all structures: monoclinic, *P*2_1_/*c*, *Z* = 4. Experiments were carried out at 150 K with Cu *K*α radiation using a Rigaku XtaLAB Synergy single-source diffractometer with a HyPix-Arc 100 detector. The absorption correction was analytical (*CrysAlis PRO*; Rigaku OD, 2023 ▸). Intensities were corrected for absorption using a multifaceted crystal model created by indexing the faces of the crystal for which data were collected (Clark & Reid, 1995 ▸). H-atom parameters were constrained.

	**1**	**2**	**3**
Crystal data			
Chemical formula	C_27_H_25_N_3_O_3_	C_21_H_17_N_3_O_5_	C_21_H_17_N_3_O_3_S_2_
*M*_r_	439.50	391.38	423.49
*a*, *b*, *c* (Å)	6.2325 (3), 22.6949 (11), 16.6898 (6)	10.3627 (4), 18.8249 (8), 10.6795 (5)	19.4242 (8), 7.2670 (3), 15.0756 (6)
β (°)	100.739 (4)	116.126 (5)	111.415 (4)
*V* (Å^3^)	2319.36 (18)	1870.46 (16)	1981.09 (15)
μ (mm^−1^)	0.67	0.84	2.68
Crystal size (mm)	0.33 × 0.03 × 0.02	0.18 × 0.03 × 0.01	0.26 × 0.1 × 0.04

Data collection			
*T*_min_, *T*_max_	0.892, 0.988	0.935, 0.991	0.652, 0.905
No. of measured, independent and observed [*I* > 2σ(*I*)] reflections	22144, 4503, 3840	17540, 3685, 3280	19584, 3961, 3417
*R*_int_	0.032	0.030	0.032
(sin θ/λ)_max_ (Å^−1^)	0.632	0.632	0.633

Refinement			
*R*[*F*^2^ > 2σ(*F*^2^)], *wR*(*F*^2^), *S*	0.037, 0.100, 1.02	0.037, 0.091, 1.04	0.034, 0.091, 1.05
No. of reflections	4503	3685	3961
No. of parameters	303	265	337
No. of restraints	0	0	496
Δρ_max_, Δρ_min_ (e Å^−3^)	0.22, −0.20	0.26, −0.22	0.34, −0.30

Computer programs: *CrysAlis PRO* (Rigaku OD, 2023 ▸), *SHELXT2018* (Sheldrick, 2015*a* ▸), *SHELXL2019*/1 (Sheldrick 2015*b* ▸), *SHELXL2019* (Sheldrick, 2015*b* ▸) and *OLEX2* (Dolomanov *et al.*, 2009 ▸).

**Table 2:** Selected geometric parameters (°) for **1**–**3**

	**1**	**2**	**3**
C3—C1—N1—C12	105.06 (14)	35.13 (17)	70.91 (18)
C1—N1—C12—O2	25.20 (17)	133.18 (13)	24.9 (2)
C1—N1—C17/20—O3/4	145.58 (13)	12.94 (19)	145.96 (14)

**Table 3:** Selected inter­molecular distances (Å, °) for **1** *Cg* indicates a ring centroid.

	C—H	H⋯O	C⋯O	C—H⋯O
C10—H10*B*⋯O2^i^	0.98	2.49	3.2995 (17)	140
C11—H11*A*⋯O2^ii^	0.98	2.45	3.3128 (18)	146
				
	C—H	C⋯*Cg*	H⋯*Cg*	C—H⋯*Cg*
C5—H5_phen­yl_⋯*Cg*_tol­yl_^i^	0.95	3.17	3.8154 (18)	127
C8—H8_phen­yl_⋯*Cg*_tol­yl_^ii^	0.95	2.85	3.6723 (16)	146

Symmetry codes: (i) −*x* + 1, −*y* + 1, −*z* + 1; (ii) −*x* + 2, *y* − 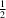, −*z* + 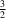.

**Table 4:** Hydrogen-bond geometry (Å, °) for **2**[Chem scheme1]

*D*—H⋯*A*	*D*—H	H⋯*A*	*D*⋯*A*	*D*—H⋯*A*
C10—H10*B*⋯O4^i^	0.98	2.61	3.418 (2)	140
C11—H11*A*⋯O4^i^	0.98	2.50	3.4478 (19)	163
C14—H14⋯O1^i^	0.95	2.35	3.197 (2)	148
C15—H15⋯O3^i^	0.95	2.64	3.318 (2)	129
C16—H16⋯O4^ii^	0.95	2.38	3.2680 (17)	156

Symmetry codes: (i) 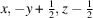; (ii) 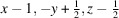.

**Table 5:** Hydrogen-bond geometry (Å, °) for **3**[Chem scheme1]

*D*—H⋯*A*	*D*—H	H⋯*A*	*D*⋯*A*	*D*—H⋯*A*
C10—H10*B*⋯O1^i^	0.98	2.71	3.600 (2)	151
C11—H11*C*⋯O1^i^	0.98	2.39	3.281 (2)	150

Symmetry code: (i) 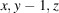.
