# Supplementary material for: Weak hy­dro­gen bonding in the structures of three double-acyl­ated amino­anti­pyrines
Source: Acta Crystallogr C Struct Chem. 2025 Nov 10;81(Pt 12):687–93. doi: 10.1107/S2053229625009581 (PMC12810325; doi:10.1107/S2053229625009581)
Supplement: Supplementary file 8 [file c-81-00687-sup8.pdf]

Acta Cryst C STRUCTURAL CHEMISTRY

Volume 81 (2025)

Supporting information for article:

Weak hydrogen bonding in the structures of three double-acylated aminoantipyrines

Lina Mardiana, Afnan B. Al Abdali, Michael J. Hall, Hamad H. Al Mamari and Paul G. Waddell

SUPPLEMENTARY INFORMATION

General Experimental Methods

All chemicals, reagents, and solvents were purchased from chemical companies (Sigma-Aldrich Chemie GmbH, Taufkirchen, Germany) and were used as received without prior purification. Reactions that required dry conditions were performed in an inert atmosphere with Ar gas. Syringes and needles for the transfer of reagents were oven-dried and cooled in a desiccator over silica gel before use. The reaction's progress was monitored by thin-layer chromatography (TLC) on glass plates pre-coated with Merck silica gel. TLC plates were examined under UV lamplight (UVGL-58 Handheld 254/365 nm). Büchi-USA rotary evaporators were used to evaporate solvents using appropriate temperatures. Flash column chromatography was performed using silica gel (Kieselgel) (70–230) mesh as an adsorbent. The purified products were characterized using NMR (^{1}H NMR, ^{13}C NMR), IR, mass spectra, and melting points. Melting points were recorded on the GallenKamp-MPd350.bm2.5 melting point apparatus (Gallenkamp, Kent, UK). Attenuated total-reflectance IR spectra were recorded on pure samples on Agilent Technologies Cary 630 FTIR (Agilent, Santa Clara, CA, USA). ^{1}H NMR spectra were recorded in $CDCl_3$ using Bruker Biospin Avance HD III 700 MHz spectrometer (Bruker, Karlsruhe, Germany). ^{1}H NMR chemical shifts (δ) were assigned in parts per million (ppm) downfield using an internal standard trimethylsilane (TMS) and were referenced to $CDCl_3$, $\delta = 7.26$. Abbreviations s, d, t, q, quin, sept, and m refer to singlet, doublet, triplet, quartet, quintet, septet, and multiplet, respectively. Chemical shifts in ^{13}C spectra (175 MHz) were quoted in ppm and referenced to the central line of the $CDCl_3$ triplet, δ C 77.0. Coupling constants (J) were recorded in hertz (Hz). GC-MS spectra were obtained using an Agilent mass spectrometer (Agilent, Santa Clara, CA, USA). Elemental analysis was performed using an EuroEA Elemental Analyzer (configuration CHN (EuroVector Instruments & Software, Milano, Italy) with a calibration type of K-factor.

N-(1,5-Dimethyl-3-oxo-2-phenyl-2,3-dihydro-1H-pyrazol-4-yl)-4-methyl-N-(4-methylbenzoyl) benzamide

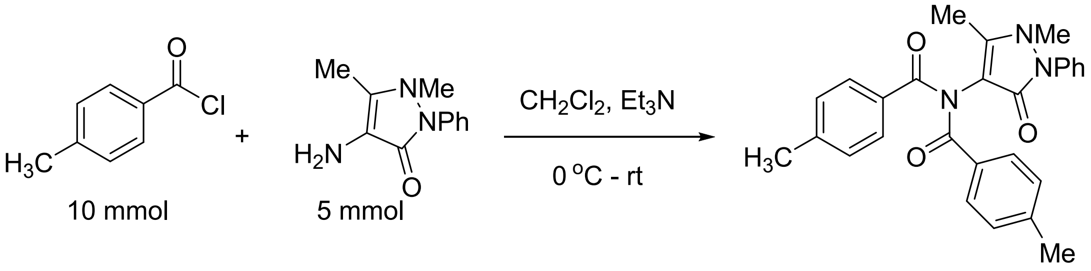

p-Toluoyl chloride (1.00 mL, 7.56 mmol) was added dropwise under an atmosphere of Ar to a cold (0 °C ice-water bath) solution of 4-aminoantipyrine (0.4935 g, 2.428 mmol) in CH_2Cl_2 (20 mL). Et_3N (1.50 mL, 10.8 mmol) was then added to the cold solution. The mixture was stirred at 0 °C for 1 h, allowed to warm up to room temperature, and then stirred for an additional 16 h. Aqueous saturated $NaHCO_3$ solution (20 mL) was added to the reaction mixture. The resulting mixture was extracted with CH_2Cl_2 (3 × 20 mL). The combined organic extracts were washed with 1 M HCl (2 × 20 mL), brine (2 × 20 mL), and water (2 × 20 mL). The organic extracts were dried over anhydrous $MgSO_4$ and filtered. Evaporation of the solvents under reduced pressure followed by recrystallization from CH_2Cl_2/petroleum ether (40-60 °C) gave the product as a white powder (0.6640 g, 62%).
Mp = 192.1-193.6 °C; ^{1}H NMR (700 MHz, $CDCl_3$) δ 7.75 (d, *J* = 8.2 Hz, 4H), 7.46–7.42 (m, 2H), 7.34–7.29 (m, 3H), 7.14 (d, *J* = 8.2 Hz, 4H), 3.05 (s, 3H), 2.34 (s, 6H), 2.07 (s, 3H); ^{13}C NMR (176 MHz, $CDCl_3$) δ 172.95, 161.49, 152.58, 143.08, 134.60, 131.80, 129.26, 129.12, 129.09, 129.05, 129.01, 127.15, 124.39, 111.68, 35.58, 21.66, 10.69; IR (solid): 3031, 2912, 1683, 1665, 1631, 1605, 1520, 1391; *m/z* (EI+) (relative intensity): 439.2 (10), 207.0 (10), 119.0 (100), 91.1(45), 56.1(20).

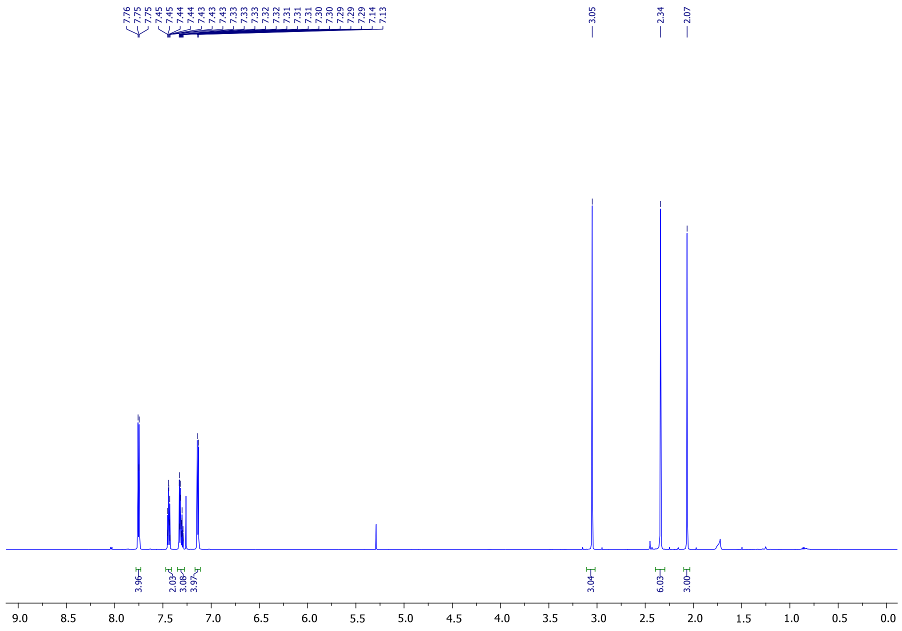

Figure S1. ^{1}H NMR of *N*-(1,5-Dimethyl-3-oxo-2-phenyl-2,3-dihydro-1*H*-pyrazol-4-yl)-4-methyl-*N*-(4-methylbenzoyl) benzamide.

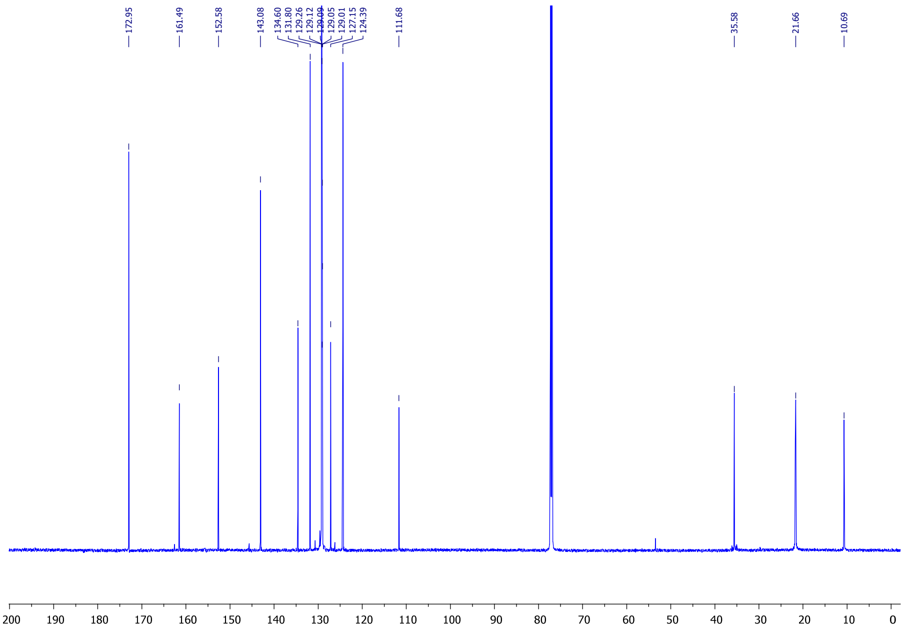

Figure S2. ^{13}C NMR of *N*-(1,5-Dimethyl-3-oxo-2-phenyl-2,3-dihydro-1*H*-pyrazol-4-yl)-4-methyl-*N*-(4-methylbenzoyl) benzamide.

Furan-2-carboxylic acid (1,5-Dimethyl-3-oxo-2-phenyl-2,3-dihydro-1*H*-pyrazol-4-yl)-furan 2-carbonyl amide

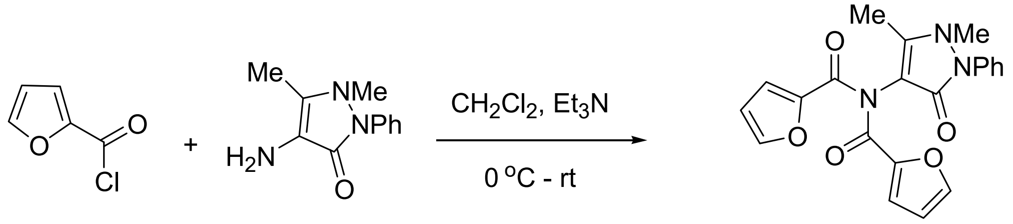

2-Furoyl chloride (0.60 mL, 6.1 mmol) was added dropwise under an atmosphere of Ar to a cold (0 °C ice-water bath) solution of 4-aminoantipyrine (0.4821 g, 2.372 mmol) in CH_2Cl_2 (20 mL). Et_3N (1.50 mL, 10.8 mmol) was then added to the cold solution. The mixture was stirred at 0 °C for 1 h, allowed to warm up to room temperature, and then stirred for an additional 16 h. Aqueous saturated $NaHCO_3$ solution (20 mL) was added to the reaction mixture. The resulting mixture was extracted with CH_2Cl_2 (3 × 20 mL). The combined organic extracts were washed with 1 M HCl (2 × 20 mL), brine (2 × 20 mL), and water (2 × 20 mL). The organic extracts were dried over anhydrous $MgSO_4$ and filtered. Evaporation of the solvents under reduced pressure followed by recrystallization from CH_2Cl_2/petroleum ether (40-60 °C) gave the product as a white powder (0.5956 g, 64%).

Mp = 174.7-176.3 °C; ^{1}H NMR (700 MHz, $CDCl_3$) δ 7.48 (dd, *J* = 1.6, 0.8 Hz, 2H), 7.47–7.43 (m, 2H), 7.38 (t, *J* = 1.6 Hz, 1H), 7.36 (d, *J* = 1.1 Hz, 1H), 7.31 (ddd, *J* = 8.6, 3.0, 0.9 Hz, 3H), 6.44 (dd, *J* = 3.6, 1.7 Hz, 2H), 3.17 (s, 3H), 2.20 (s, 3H); ^{13}C NMR (176 MHz, $CDCl_3$) δ 161.41, 160.70, 152.95, 147.25, 146.58, 134.48, 129.30, 127.24, 124.53, 119.43, 112.30, 109.29, 35.56, 10.90; IR (solid): 3138, 2914, 1702, 1691, 1673, 1627, 1580, 1513, 1448, 1402; *m/z* (EI+) (relative intensity): 391.1 (10), 207.0 (10), 95.0 (100), 91.1(45), 56.1 (45).

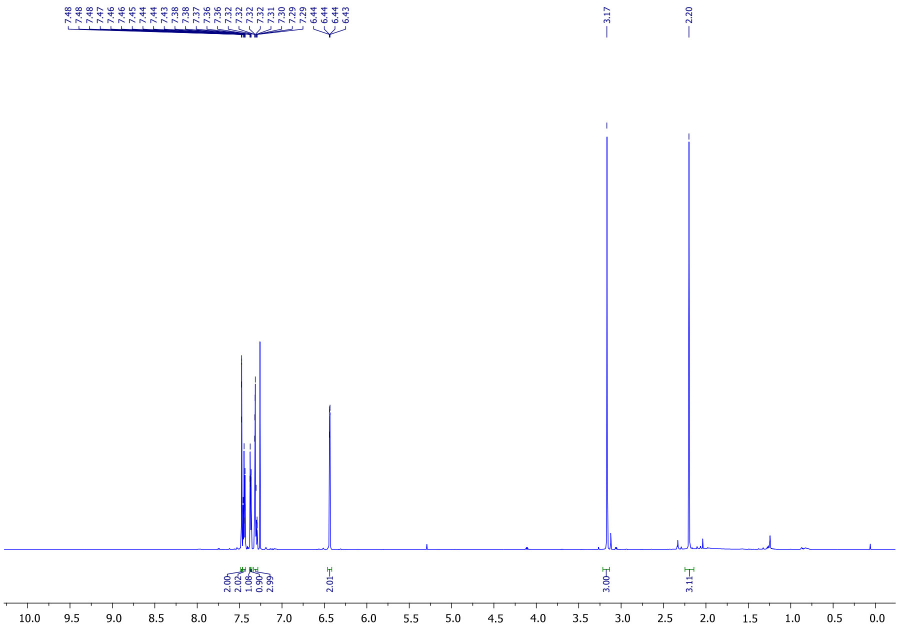

Figure S3. ^{1}H NMR of Furan-2-carboxylic acid (1,5-Dimethyl-3-oxo-2-phenyl-2,3-dihydro-1*H*-pyrazol-4-yl)-furan 2-carbonyl amide.

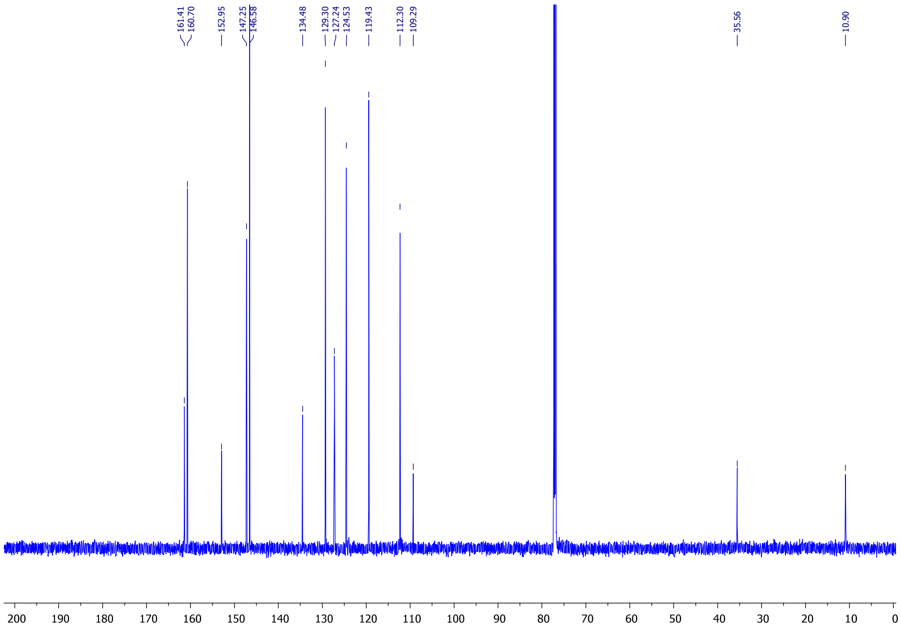

Figure S4. ^{13}C NMR of Furan-2-carboxylic acid (1,5-Dimethyl-3-oxo-2-phenyl-2,3-dihydro-1*H*-pyrazol-4-yl)-furan 2-carbonyl amide.

Thiophene-2-carboxylic acid (1,5-Dimethyl-3-oxo-2-phenyl-2,3-dihydro-1*H*-pyrazol-4-yl)-thiophene 2-carbonyl amide

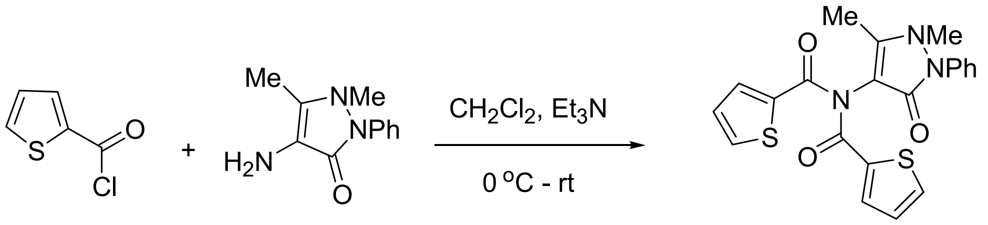

2-Thiophenecarbonnyl chloride (0.80 mL, 7.5 mmol) was added dropwise under an atmosphere of Ar to a cold (0 °C ice-water bath) solution of 4-aminoantipyrine (0.5024 g, 2.472 mmol) in CH_2Cl_2 (20 mL). Et_3N (1.50 mL, 10.8 mmol) was then added to the cold solution. The mixture was stirred at 0 °C for 1 h, allowed to warm up to room temperature, and then stirred for an additional 16 h. Aqueous saturated $NaHCO_3$ solution (20 mL) was added to the reaction mixture. The resulting mixture was then extracted with CH_2Cl_2 (3 × 20 mL). The combined organic extracts were washed with 1 M HCl (2 × 20 mL), brine (2 × 20 mL), and water (2 × 20 mL). The organic extracts were dried over anhydrous $MgSO_4$ and filtered. Evaporation of the solvents under reduced pressure followed by recrystallization from CH_2Cl_2/petroleum ether (40-60 °C) gave the product as a white powder (0.5347 g, 52%).

Mp = 178.2-179.0 °C; ^{1}H NMR (700 MHz, $CDCl_3$) δ 7.87 (dd, J = 3.8, 1.2 Hz, 2H), 7.58–7.53 (m, 2H), 7.49–7.43 (m, 2H), 7.40–7.34 (m, 2H), 7.34–7.30 (m, 1H), 7.02 (dd, J = 5.0, 3.8 Hz, 2H), 3.19 (s, 3H), 2.22 (s, 3H); ^{13}C NMR (176 MHz, $CDCl_3$) δ 165.70, 161.53, 153.29, 136.99, 134.43, 134.40, 134.38, 133.50, 129.36, 127.85, 127.81, 127.40, 124.67, 110.00, 35.40, 10.83; IR (solid): 3079, 2935, 1681, 1668, 1653, 1605, 1533, 1443; *m/z* (EI+) (relative intensity): 423.0 (10), 281 (10), 207.0 (30), 111.0 (100), 96.0 (5), 73.1 (5), 56.1 (20).

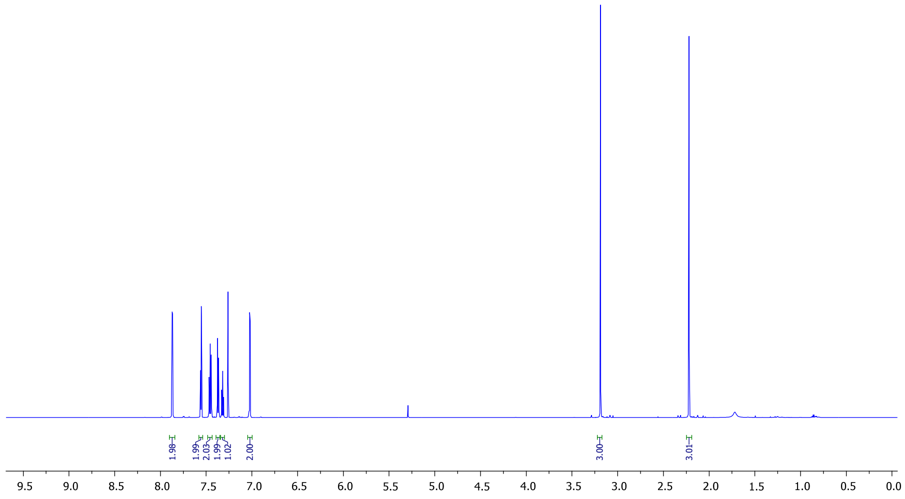

Figure S5. ^{1}H NMR of Thiophene-2-carboxylic acid (1,5-Dimethyl-3-oxo-2-phenyl-2,3-dihydro-1*H*-pyrazol-4-yl)-thiophene 2-carbonyl amide.

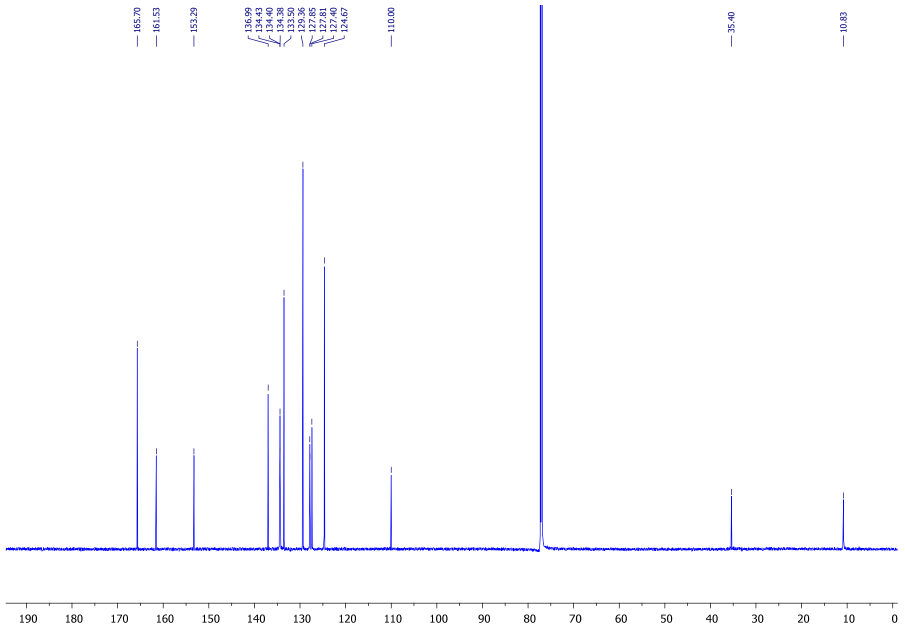

Figure S6. ^{1}H NMR of Thiophene-2-carboxylic acid (1,5-Dimethyl-3-oxo-2-phenyl-2,3-dihydro-1*H*-pyrazol-4-yl)-thiophene 2-carbonyl amide.

Crystal growth by ENaCt

Using a STP Labtech Mosquito Liquid-Handling Robot, 200 nL of one of four oils (PDMSO, FC-40, FY and mineral oil) was dispensed onto a 96-well SWISSCI LCP plate with a 100-micron spacer. 50 nL of stock solution, containing the compound of interest, was collected from the parent plate and dispensed into the oil droplets within the wells. The plates were sealed with a glass cover slip and stored in the dark at room temperature. After 2 weeks, evaluation of crystal growth was carried out visually by cross-polarised optical microscopy.

ENaCt compound 1

Preparation of stock solutions

Compound **1** (12 mg) was weighed into a glass vial (50 x 12 mm), followed by the addition of $CHCl_3$ (600 µL) to give a clear solution. The solution was then split equally into 12 x 1.75 mL screw top glass vials. Samples were then air-dried in a fume hood, giving ~ 1 mg of compound **1** in each vial. Stock solutions were prepared as follows. Solvent was added into each vial in 12 µL portions until the material was fully dissolved, up to a maximum of 192 µL. Where the samples were not fully soluble, the supernatant was used.

Vial	Solvent	Mass of compound/ mg	Volume of solvent / µL	Concentration of substrate/ mg mL^{-1}
1	DMSO	1	12	83
2	DMF	1	12	83
3	MeOH	1	96	10
4	2,2,2-TFE	1	12	83
5	Toluene	1	96	10
6	1,2-DCE	1	12	83
7	2-MeTHF	1	192	Supernatant
8	1,4-Dioxane	1	12	83
9	EtOAc	1	192	Supernatant
10	MeCN	1	12	83
11	MIBK	1	192	Supernatant
12	NM	1	12	83

Table S1. Stock solution preparation for compound **1**.

ENaCt experiments and results

Compound 1_P1														
Vial	Standard Method		Volume of Oil = 200 nL											
	Volume of Solvent		50 nL											
	Solvents		1	2	3	4	5	6	7	8	9	10	11	12
1	DMSO	A	No oil	PDMSO					No oil	FY				
	DMSO	B	No oil	FC-40					No oil	MO				
2	DMF	C	No oil	PDMSO					No oil	FY				
	DMF	D	No oil	FC-40					No oil	MO				
3	MeOH	E	No oil	PDMSO					No oil	FY				
	MeOH	F	No oil	FC-40					No oil	MO				
4	2,2,2-TFE	G	No oil	PDMSO					No oil	FY				
	2,2,2-TFE	H	No oil	FC-40					No oil	MO				

Compound 1_P1														
Vial	Standard Method		Volume of Oil = 200 nL											
	Volume of Solvent		50 nL											
	Solvents		1	2	3	4	5	6	7	8	9	10	11	12
1	DMSO	A	3	4	4	4	4	4	3	3	4	4	4	4
	DMSO	B	3	4	4	4	3	3	3	3	3	3	4	4
2	DMF	C	2	2	2	2	2	2	2	2	2	2	2	2
	DMF	D	2	2	2	2	2	2	2	2	2	2	2	2
3	MeOH	E	2	2	2	2	2	2	2	2	2	2	2	2
	MeOH	F	2	2	2	2	2	2	2	2	2	2	2	2
4	2,2,2-TFE	G	2	2	2	2	2	2	2	2	2	2	2	2
	2,2,2-TFE	H	2	2	2	2	2	2	2	2	2	2	2	2

Compound 1_P2														
Vial	Standard Method		Volume of Oil = 200 nL											
	Volume of Solvent		50 nL											
	Solvents		1	2	3	4	5	6	7	8	9	10	11	12
5	Tol	A	No oil	PDMSO					No oil	FY				
	Tol	B	No oil	FC-40					No oil	MO				
6	1,2-DCE	C	No oil	PDMSO					No oil	FY				
	1,2-DCE	D	No oil	FC-40					No oil	MO				
7	2-MeTHF	E	No oil	PDMSO					No oil	FY				
	2-MeTHF	F	No oil	FC-40					No oil	MO				
8	1,4-Dioxane	G	No oil	PDMSO					No oil	FY				
	1,4-Dioxane	H	No oil	FC-40					No oil	MO				

Compound 1_P2														
Vial	Standard Method		Volume of Oil = 200 nL											
	Volume of Solvent		50 nL											
	Solvents		1	2	3	4	5	6	7	8	9	10	11	12
5	Tol	A	2	2	2	2	2	2	2	2	2	2	2	2
	Tol	B	2	2	2	2	2	2	2	2	2	2	2	2
6	DCE	C	2	2	2	2	2	2	2	2	2	2	2	2
	DCE	D	2	2	2	2	2	2	2	2	2	2	2	2
7	2-MeTHF	E	2	2	2	2	2	2	2	3	3	3	3	3
	2-MeTHF	F	2	2	2	2	2	2	2	3	3	3	3	3
8	1,4-Dioxane	G	2	2	2	2	2	2	2	2	2	2	2	2
	1,4-Dioxane	H	2	2	2	2	2	2	2	2	2	2	2	2

Compound 1_P3														
Vial	Standard Method		Volume of Oil = 200 nL											
	Volume of Solvent		50 nL											
	Solvents		1	2	3	4	5	6	7	8	9	10	11	12
9	EtOAc	A	No oil	PDMSO					No oil	FY				
	EtOAc	B	No oil	FC-40					No oil	MO				
10	MeCN	C	No oil	PDMSO					No oil	FY				
	MeCN	D	No oil	FC-40					No oil	MO				
11	MIBK	E	No oil	PDMSO					No oil	FY				
	MIBK	F	No oil	FC-40					No oil	MO				
12	NM	G	No oil	PDMSO					No oil	FY				
	NM	H	No oil	FC-40					No oil	MO				

Compound 1_P3														
Vial	Standard Method		Volume of Oil = 200 nL											
	Volume of Solvent		50 nL											
	Solvents		1	2	3	4	5	6	7	8	9	10	11	12
9	EtOAc	A	2	2	2	2	2	2	2	2	2	2	2	2
	EtOAc	B	2	2	2	2	2	2	2	2	2	2	2	2
10	MeCN	C	2	2	4	2	2	2	2	2	2	2	2	2
	MeCN	D	2	2	2	2	2	2	2	2	2	2	2	2
11	MIBK	E	2	2	2	2	2	2	2	2	2	2	2	2
	MIBK	F	2	2	2	2	2	2	2	2	2	2	2	2
12	NM	G	2	2	2	2	2	2	2	2	2	2	2	2
	NM	H	2	2	2	2	2	2	2	2	2	2	2	2

Figure S7. ENaCt plate layouts and crystallisation outcomes based on examination by cross-polarised optical microscopy. Results are classified as 1 – remains in solution, 2 – oil or amorphous solid, 3 – microcrystalline, 4 – crystals suitable for SCXRD.

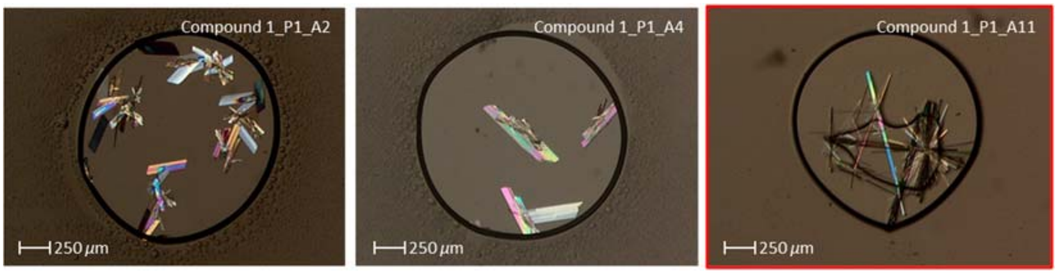

Figure S8. Cross-polarised optical microscopy images of selected ENaCt wells, classified as suitable for SCXRD. The image highlighted in red indicates the sample from which SCXRD data was obtained.

ENaCt compound 2

Preparation of stock solutions

Compound **2** (12 mg) was weighed into a glass vial (50 x 12 mm), followed by the addition of $CHCl_3$ (600 µL) to give a solution. The solution was then split equally into 12 x 1.75 mL screw top glass vials. Samples were then air-dried in a fume hood, giving ~ 1 mg of compound **2** in each vial.

Stock solutions were prepared as follows. Solvent was added into each vial in 12 µL portions until the material was fully dissolved, up to a maximum of 192 µL. Where the samples were not fully soluble, the supernatant was used.

Vial	Solvent	Mass of compound/ mg	Volume of solvent added/ µL	Concentration of substrate/ mg mL^{-1}
1	DMSO	1	48	21
2	DMF	1	48	21
3	MeOH	1	192	Supernatant
4	2,2,2-TFE	1	24	42
5	Toluene	1	192	Supernatant
6	1,2-DCE	1	192	Supernatant
7	2-MeTHF	1	192	Supernatant
8	1,4-Dioxane	1	192	Supernatant
9	EtOAc	1	192	Supernatant
10	MeCN	1	192	5.2
11	MIBK	1	192	Supernatant
12	NM	1	48	21

Table S2. Stock solution preparation for compound **2**.

ENaCt experiments and results

Compound 2_P1														
Vial	Standard Method		Volume of Oil = 200 nL											
	Volume of Solvent		50 nL											
	Solvents		1	2	3	4	5	6	7	8	9	10	11	12
1	DMSO	A	No oil	PDMSO					No oil	FY				
	DMSO	B	No oil	FC-40					No oil	MO				
2	DMF	C	No oil	PDMSO					No oil	FY				
	DMF	D	No oil	FC-40					No oil	MO				
3	MeOH	E	No oil	PDMSO					No oil	FY				
	MeOH	F	No oil	FC-40					No oil	MO				
4	2,2,2-TFE	G	No oil	PDMSO					No oil	FY				
	2,2,2-TFE	H	No oil	FC-40					No oil	MO				

Compound 2_P1														
Vial	Standard Method		Volume of Oil = 200 nL											
	Volume of Solvent		50 nL											
	Solvents		1	2	3	4	5	6	7	8	9	10	11	12
1	DMSO	A	4	2	2	2	2	2	4	3	2	3	2	3
	DMSO	B	3	1	3	3	3	3	3	3	3	3	1	1
2	DMF	C	2	2	2	2	2	2	2	2	2	2	2	2
	DMF	D	2	3	2	2	2	2	2	2	2	2	2	2
3	MeOH	E	2	2	2	2	2	2	2	2	2	2	3	2
	MeOH	F	2	2	2	2	2	2	2	2	2	2	2	2
4	2,2,2-TFE	G	2	2	2	2	2	2	2	2	2	2	2	2
	2,2,2-TFE	H	2	2	2	2	2	2	2	2	2	2	2	2

Compound 2_P2														
Vial	Standard Method		Volume of Oil = 200 nL											
	Volume of Solvent		50 nL											
	Solvents		1	2	3	4	5	6	7	8	9	10	11	12
5	Tol	A	No oil	PDMSO					No oil	FY				
	Tol	B	No oil	FC-40					No oil	MO				
6	1,2-DCE	C	No oil	PDMSO					No oil	FY				
	1,2-DCE	D	No oil	FC-40					No oil	MO				
7	2-MeTHF	E	No oil	PDMSO					No oil	FY				
	2-MeTHF	F	No oil	FC-40					No oil	MO				
8	1,4-Dioxane	G	No oil	PDMSO					No oil	FY				
	1,4-Dioxane	H	No oil	FC-40					No oil	MO				

Compound 2_P2														
Vial	Standard Method		Volume of Oil = 200 nL											
	Volume of Solvent		50 nL											
	Solvents		1	2	3	4	5	6	7	8	9	10	11	12
5	Tol	A	2	2	2	2	2	2	2	2	2	2	2	2
	Tol	B	2	2	2	2	2	2	2	2	2	2	2	2
6	1,2-DCE	C	2	2	2	2	2	2	2	2	2	2	2	2
	1,2-DCE	D	2	2	2	2	2	2	2	2	2	3	2	2
7	2-MeTHF	E	2	2	2	2	2	2	2	2	2	2	2	2
	2-MeTHF	F	2	2	2	2	2	2	2	2	2	2	2	2
8	1,4-Dioxane	G	2	2	2	2	2	2	2	2	2	2	2	2
	1,4-Dioxane	H	2	2	2	2	2	2	2	2	2	2	2	2

Compound 2_P3														
Vial	Standard Method		Volume of Oil = 200 nL											
	Volume of Solvent		50 nL											
	Solvents		1	2	3	4	5	6	7	8	9	10	11	12
9	EtOAc	A	No oil	PDMSO					No oil	FY				
	EtOAc	B	No oil	FC-40					No oil	MO				
10	MeCN	C	No oil	PDMSO					No oil	FY				
	MeCN	D	No oil	FC-40					No oil	MO				
11	MIBK	E	No oil	PDMSO					No oil	FY				
	MIBK	F	No oil	FC-40					No oil	MO				
12	NM	G	No oil	PDMSO					No oil	FY				
	NM	H	No oil	FC-40					No oil	MO				

Compound 2_P3														
Vial	Standard Method		Volume of Oil = 200 nL											
	Volume of Solvent		50 nL											
	Solvents		1	2	3	4	5	6	7	8	9	10	11	12
9	EtOAc	A	2	2	2	2	2	2	2	2	2	2	2	2
	EtOAc	B	2	2	2	2	2	2	2	2	2	2	2	2
10	MeCN	C	2	2	2	2	2	2	2	2	2	2	3	2
	MeCN	D	2	2	2	2	2	2	2	2	2	3	2	2
11	MIBK	E	2	2	2	2	2	2	2	2	2	2	2	2
	MIBK	F	2	2	2	2	2	2	2	2	2	2	2	2
12	NM	G	2	2	2	2	2	2	2	2	2	2	2	2
	NM	H	2	2	2	2	2	2	2	2	2	2	2	2

Figure S9. ENaCt plate layouts and crystallisation outcomes based on examination by cross-polarised optical microscopy. Results are classified as 1 – remains in solution, 2 – oil or amorphous solid, 3 – microcrystalline, 4 – crystals suitable for SCXRD.

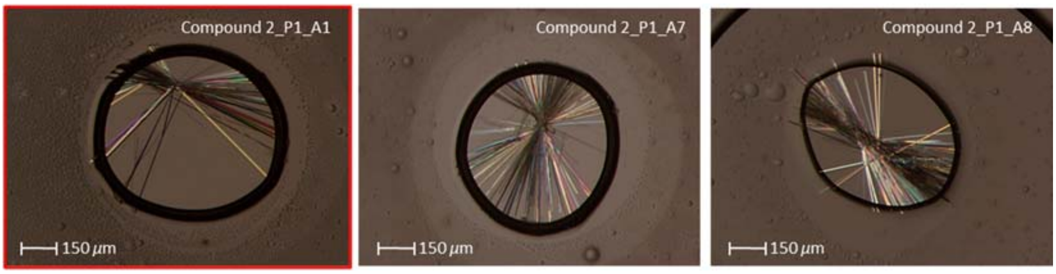

Figure S10. Cross-polarised optical microscopy images of selected ENaCt wells, classified as suitable for SCXRD. The image highlighted in red indicates the sample from which SCXRD data was obtained.

ENaCt compound 3

Preparation of stock solutions

Compound **3** (18.8 mg) was weighed into a glass vial (50 x 12 mm), followed by the addition of $CHCl_3$ (600 µL) to give a solution. The solution was then split equally into 12 x 1.75 mL screw top glass vials. Samples were then air-dried in a fume hood, giving ~ 1.6 mg of compound **3** in each vial. Stock solutions were prepared as follows. Solvent was added into each vial in 12 µL portions until the material was fully dissolved, up to a maximum of 192 µL. Where the samples were not fully soluble, the supernatant was used.

Vial	Solvent	Mass of compound/ mg	Volume of solvent / µL	Concentration of substrate/ mg mL^{-1}
1	DMSO	1.6	24	67
2	DMF	1.6	12	133
3	MeOH	1.6	192	Supernatant
4	2,2,2-TFE	1.6	12	133
5	Toluene	1.6	192	Supernatant
6	1,2-DCE	1.6	24	67
7	2-MeTHF	1.6	192	Supernatant
8	1,4-Dioxane	1.6	192	5.2
9	EtOAc	1.6	192	Supernatant
10	MeCN	1.6	48	33
11	MIBK	1.6	192	Supernatant
12	NM	1.6	48	33

Table S3. Stock solution preparation for compound **3**.

ENaCt experiments and results

Compound 3_P1														
Vial	Standard Method		Volume of Oil = 200 nL											
	Volume of Solvent		50 nL											
	Solvents		1	2	3	4	5	6	7	8	9	10	11	12
1	DMSO	A	No oil	PDMSO					No oil	FY				
	DMSO	B	No oil	FC-40					No oil	MO				
2	DMF	C	No oil	PDMSO					No oil	FY				
	DMF	D	No oil	FC-40					No oil	MO				
3	MeOH	E	No oil	PDMSO					No oil	FY				
	MeOH	F	No oil	FC-40					No oil	MO				
4	2,2,2-TFE	G	No oil	PDMSO					No oil	FY				
	2,2,2-TFE	H	No oil	FC-40					No oil	MO				

Compound 3_P1														
Vial	Standard Method		Volume of Oil = 200 nL											
	Volume of Solvent		50 nL											
	Solvents		1	2	3	4	5	6	7	8	9	10	11	12
1	DMSO	A	4	4	4	4	3	2	4	4	4	4	4	4
	DMSO	B	4	4	4	4	4	4	4	4	4	4	4	4
2	DMF	C	1	1	1	1	1	1	1	2	2	2	2	2
	DMF	D	1	2	1	1	1	1	1	1	1	1	1	1
3	MeOH	E	2	2	2	2	2	2	2	2	2	2	2	2
	MeOH	F	2	2	2	2	2	2	2	2	2	2	2	2
4	2,2,2-TFE	G	1	1	1	1	1	1	1	1	1	2	2	1
	2,2,2-TFE	H	1	1	1	1	1	1	1	1	1	1	1	1

Compound 3_P2														
Vial	Standard Method		Volume of Oil = 200 nL											
	Volume of Solvent		50 nL											
	Solvents		1	2	3	4	5	6	7	8	9	10	11	12
5	Tol	A	No oil	PDMSO					No oil	FY				
	Tol	B	No oil	FC-40					No oil	MO				
6	1,2-DCE	C	No oil	PDMSO					No oil	FY				
	1,2-DCE	D	No oil	FC-40					No oil	MO				
7	2-MeTHF	E	No oil	PDMSO					No oil	FY				
	2-MeTHF	F	No oil	FC-40					No oil	MO				
8	1,4-Dioxane	G	No oil	PDMSO					No oil	FY				
	1,4-Dioxane	H	No oil	FC-40					No oil	MO				

Compound 3_P2														
Vial	Standard Method		Volume of Oil = 200 nL											
	Volume of Solvent		50 nL											
	Solvents		1	2	3	4	5	6	7	8	9	10	11	12
5	Tol	A	1	3	3	3	3	3	1	1	1	1	1	1
	Tol	B	1	4	3	1	3	3	2	3	3	3	3	3
6	DCE	C	1	3	2	3	3	2	1	1	1	1	1	1
	DCE	D	3	1	4	1	4	4	3	3	2	3	3	2
7	2-MeTHF	E	2	3	2	2	2	2	2	2	2	2	4	2
	2-MeTHF	F	2	2	2	2	2	2	2	3	2	3	2	2
8	1,4-Dioxane	G	1	2	2	2	2	2	1	2	2	2	2	2
	1,4-Dioxane	H	1	2	2	2	2	2	2	3	2	3	2	2

Compound 3_P3															
Vial	Standard Method		Volume of Oil = 200 nL												
	Volume of Solvent		50 nL												
	Solvents		1	2	3	4	5	6	7	8	9	10	11	12
9	EtOAc	A	No oil	PDMSO					No oil	FY				
	EtOAc	B	No oil	FC-40					No oil	MO				
10	MeCN	C	No oil	PDMSO					No oil	FY				
	MeCN	D	No oil	FC-40					No oil	MO				
11	MIBK	E	No oil	PDMSO					No oil	FY				
	MIBK	F	No oil	FC-40					No oil	MO				
12	NM	G	No oil	PDMSO					No oil	FY				
	NM	H	No oil	FC-40					No oil	MO				

Compound 3_P3															
Vial	Standard Method		Volume of Oil = 200 nL												
	Volume of Solvent		50 nL												
	Solvents		1	2	3	4	5	6	7	8	9	10	11	12
9	EtOAc	A	1	2	3	2	2	3	1	1	4	1	4	4
	EtOAc	B	2	2	2	3	3	1	1	3	3	3	2	3
10	MeCN	C	1	2	2	2	2	2	1	2	2	2	2	2
	MeCN	D	2	2	4	2	2	2	3	2	3	2	2	2
11	MIBK	E	1	2	2	2	2	2	1	3	2	2	2	2
	MIBK	F	1	2	2	2	2	2	1	2	2	2	2	2
12	NM	G	1	1	1	1	1	1	1	2	1	1	1	1
	NM	H	1	2	2	2	2	2	1	2	2	2	2	2

Figure S11. ENaCt plate layouts and crystallisation outcomes based on examination by cross-polarised optical microscopy. Results are classified as 1 – remains in solution, 2 – oil or amorphous solid, 3 – microcrystalline, 4 – crystals suitable for SCXRD.

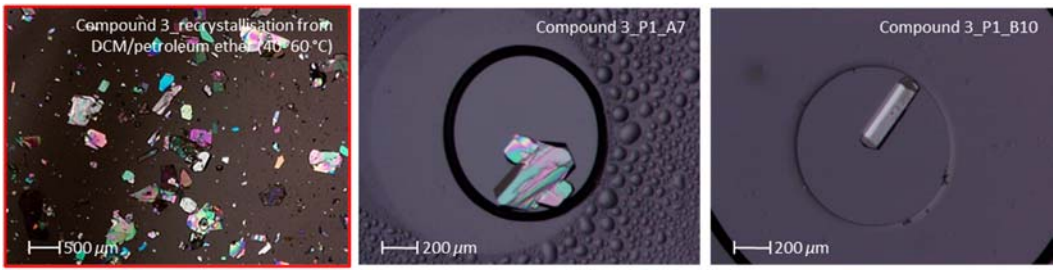

Figure S12. Cross-polarised optical microscopy images of selected ENaCt wells, classified as suitable for SCXRD, and a sample formed through classical crystallisation from DCM/petroleum ether (40-60 °C). The image highlighted in red indicates the sample from which SCXRD data was obtained.
